# Funding Opportunities Designed to Promote Antiracist Change Across a Health Sciences University

**DOI:** 10.1001/jamanetworkopen.2023.37096

**Published:** 2023-10-10

**Authors:** Constance R. Tucker, Elizabeth Lahti, Patricia A. Carney

**Affiliations:** 1 Academic Affairs, Provost Office, Oregon Health & Science University, Portland; 2 Division of Hospital Medicine, School of Medicine, Oregon Health & Science University, Portland

## Abstract

**Importance:**

As health professionals acknowledge the historic and current influences racism has on patient care and health outcomes, leaders must develop antiracist activities to disrupt current narratives.

**Objective:**

To examine the outcomes of antiracism funding opportunities for development, implementation, and evaluation of initiatives across a multi–health professions academic university.

**Design, Setting, and Participants:**

This mixed-methods cohort study involved the evaluation of 17 antiracism projects conducted at a midsize single academic university in the Pacific Northwest with schools of dentistry, medicine, nursing, pharmacy, and public health. Projects were designed by students, faculty, staff, and community members, many of whom were from diverse backgrounds. Data collection and analyses were conducted between January and December 2022.

**Main Outcomes and Measures:**

Influence and reach of institutionally funded antiracist projects and best practices for funding them. Key metrics included project types, allocation of funds, level of community engagement, number of individuals engaged, demographic characteristics of project leaders, and project facilitators and barriers.

**Results:**

Forty-two proposals were submitted, and 17 were selected for funding, representing 3 of 5 health profession schools. Study teams included women (15 of 19 [79%]), gender queer (1 [5%]), Asian American (5 [26%]), Black or African American (2 [11%]), Hispanic or Latinx (1 [5%]), Middle Eastern, North African, or biracial (4 [21%]) and White (7 [37%]) individuals. Four of 17 teams (24%) returned funds because their proposed projects exceeded their workload. Eight projects (47%) were fully implemented, most in the School of Medicine. Community engagement surveys were completed by 10 projects (59%), and 1741 participants engaged in 1 or more funded events. Two focus groups were attended by 7 of 17 projects (41%), which reflected diversity among grantees. Participants noted that facilitators of antiracist activities included strong community engagement, improved well-being, and sense of support by both the community and the institution. Barriers to advancing antiracist initiatives included restrictive timelines, being unprepared for the time needed for their efforts, distrust from community members due to previous experiences with diversity and inclusion projects, and difficulties navigating complex institutional systems and processes.

**Conclusions and Relevance:**

The findings of this study suggest that efforts needed to implement antiracist change should not be underestimated. Engagement should be inclusive across academic health centers and communities. Future efforts need to support innovator full-time equivalent support, individual mentorship, and institutional sponsorship.

## Key Points

**Question:**

How can health professions educators utilize principles of antiracism and disruptive innovation to become transformative agents for antiracist change?

**Findings:**

In this cohort study involving 17 funding opportunities from a multischool academic health center, over half the projects were implemented, engaging a total of 1741 participants. Participants in focus groups reported better well-being and described institutional catalysts and barriers to stimulating and sustaining antiracist programs designed to change behaviors.

**Meaning:**

In this study, robust planning and implementation of antiracist funding opportunities were associated with individual stakeholder engagement and improved participant wellness; however, these initiatives require institutional flexibility, intention, advocacy, and ongoing support.

## Teaser

This cohort study examines the outcomes of antiracism funding opportunities for development, implementation, and evaluation of initiatives across a health sciences university in the United States.

## Introduction

Health professions education, particularly at predominately White institutions, continues to operate with a legacy of racism, causing ongoing harm, reinforcing race as a biological construct, and creating ongoing inertia that sustains the status quo for admissions and hiring processes and policies.^1^ These issues are further escalated when learners’ and patients’ examinations and evaluations continue to magnify structural discrimination in assessments.^1,2,3,4,5,6^ This stained history reinforces Dr Ibram X. Kendi’s words that “it is racist power that creates racist policies”^7^ that lead to racial inequities. In response to these structural inequities, numerous efforts, at both individual and institutional levels, are being created and implemented to counteract racist ideas, thoughts, and policies.

An additional framework that informs the institutional response to antiracism is the idea of disruptive innovation.^8^ Disruptive innovations are approaches with little to no support early on that steadily move into the environment and reshape the institution or industry. They need time to fully develop as their traction can be slow and tricky, but they survive and then thrive over dominant approaches. These disrupters often persist in models that are not well integrated into current institutional structures. They often have a technological enabler and a business model and are of economic value to the overall community. Telehealth, genomic technology, and 3-dimensional printing are all examples of disruptive innovations.^9^ Here, we argue that disruptive innovators have the potential to be transformative agents of antiracist change that to date are not well understood in health professions education.

Kendi^7^ recommends antiracist approaches where institutions can critically examine and then implement changes to address racism in their procedures, policies, and practices. A final framework to inform antiracist work includes the 9 recommendations from Argueza et al^1^ to (1) commit to antiracism in mission, vision, and values; (2) conduct a systematic assessment of institutional policies, procedures, and norms; (3) collect, report, and respond to data on racial inequities; (4) invest funding and other resources to support antiracism initiatives; (5) avoid placing the burden of change on people of color; (6) share and yield decision-making power; (7) intentionally address racism; (8) teach how to and then expect everyone to practice antiracism; and (9) mobilize allies to leverage their privilege positively.^1^

To draw from lived experiences of individuals within our own institution and current understanding of best practices in antiracist work, we aligned with what Camara P. Jones calls a “see, name, understand, and act”^10^ antiracist framework and proposed the idea of Racial, Equity, and Inclusion (REI) Mini-Funding Opportunities to support projects specifically designed to promote antiracism across all of Oregon Health & Science University’s (OHSU’s) mission areas.^1,11^

## Methods

### Study Setting and Initiative Development

OHSU is Oregon’s only academic health center, which educates many of Oregon’s future health professionals and scientists in dentistry, medicine, nursing, pharmacy, and public health. In June 2020, in response to the murder of George Floyd in Minneapolis, the OHSU Center for Diversity and Inclusion formed a workgroup to encourage initiatives that address systematic racism and disparities present on the OHSU campus. Rather than look to individuals outside of the institution to create curricula, the workgroup tapped into a deep well of expertise among faculty, staff, and students already working within the institution. In alignment with the fourth recommendation from Argueza et al,^1^ Kendi’s antiracist approach,^7^ and the approach to disruptive innovation from Christensen et al,^8^ $200 000 was allocated to support ideas that address OHSU’s inherent structural racism. The intent of offering REI funding to individuals within the organization was to reduce the burden often placed on minoritized faculty (eg, “minority tax”) while increasing institutional valuation and stimulating and sustaining antiracist behavior at OHSU.^12^

The design and implementation of OHSU’s REI funding initiative was exploratory in nature, while contributing to the field in innovative ways.^13^ From beginning to end, we included a novel design for the call for proposals, a transparent selection process using a decision-making rubric, an intentionally diverse selection committee, and subsequent mentoring and coaching to ensure success. The program’s structure utilized innovative approaches to garner broad and diverse participation from inside and outside the institution. For example, to utilize principles of antiracism^7^ and be broadly inclusive, the call for submissions was intentionally titled a “funding opportunity” rather than a grant opportunity. It was translated into 6 different languages and emphasized transparency in selection through a prepublished rubric promoting the intersectionality of identities and multiple layers of inequality.^14^ It also fostered partnership and collaboration with diverse community groups or established internal employee and learner resource groups. Lastly, it supported identifying and pairing awardees with an institutional sponsor to ensure that selected proposals would receive coaching and support to navigate barriers.

The goals of the REI Mini-Funding opportunity program were to (1) encourage faculty, staff, and students to be actively involved in reducing racism and the intersectionality of race and sexism, race and classism, race and homophobia, race and ageism, and race and ableism; 2) promote antiracist practices in recruitment and retention efforts in educational programs at OHSU; and 3) support the development of educational initiatives and policies that value humanity, promote antiracism, and allow all voices to be recognized, heard, and valued. While this work may be unique to one academic health center, we believe the lack of disruptive innovations in antiracist work is global; thus, the processes and outcomes of this project provide insights for other academic health institutions.

### Request for Applications and Selection Process

We reviewed existing literature, with a diversity, equity, inclusion, and antiracism lens to determine submission and selection criteria, and recommend program evaluation approaches for program proposals (eAppendix in Supplement 1). A call for REI Mini-Funding applications went out to all institutional faculty, staff, and learners in October 2021. Applications were reviewed by an intentionally diverse and interdisciplinary group of internal and external partners. Awardees were selected based on meeting criteria outlined in the selection rubric and were notified in December 2021. Of note, several awardees proposed similar projects and were encouraged to collaborate toward combining their proposal into a single submission.

### Design and Measurement Approach

This was a cohort study with a mixed-methods approach that included administering surveys (quantitative approach) to and conducting focus groups with recipients of OHSU’s REI Mini-Funding opportunity, at 4 different points over a 12-month period (January to December 2022): first, upon receipt of funding (pre/time 1); second, 2 months after receipt of funding (early/time 2); third, 6 months after receipt of funding (mid/time 3); and, finally, 11 months after receipt of funding (final/time 4). All study activities were reviewed and approved by OHSU’s institutional review board. Informed consent was obtained from all study participants during Time 1 of the study. We followed Strengthening the Reporting of Observational Studies in Epidemiology (STROBE) reporting guidelines when developing this manuscript.^15^

At time 1, we assessed demographic and other characteristics of the study team by having a designated team member complete a survey that asked about role on the project, gender identity, sexual orientation, age, racial identity, primary language, disability, and institutional affiliation of the study participants. At time 2, recipients completed a Community Engagement Survey (CES) that included 4 questions to determine how each project intended to engage participants. The survey questions examined project participants’ demographics, institutional mission area, level of engagement, and narrative comments. The community engagement framework was developed internally at OHSU.^16^ First, we defined community *partners* as OHSU faculty, staff, learners, patients and visitors, and the community at large. Second, the institutional mission was identified as research, education, clinical, and community. Third, we queried each recipient about their approach and level of engagement used in their project. Levels of engagement included hierarchical approaches from lower levels of providing information and consultation with community partners to higher levels, such as collaboration and enabling. This approach provided authors with insights into how participants designed their projects in collaboration with community partners, rather than informing community partners of a project without their active participation. Finally, the survey allowed for narrative comments about each project’s intended influence.

At time 3, we conducted focus groups, which lasted approximately 1 hour. Focus group questions were developed by study investigators to assess (1) REI-funded program evaluation and (2) program leader well-being and purpose. Focus groups were facilitated by 2 qualitative researchers who were not the principal investigators of the study or recipients of any funding, with each one taking the role as lead facilitator for each session. One researcher took field notes during each focus group session.

At time 4, participants submitted a final report that described their activities and evaluations undertaken, while also accounting for the use of funds. The report included 8 open-ended questions that were aligned with the first objective of the study, ie, to identify how faculty, staff, and learners engage in reducing racism and the intersectionality of race and sexism, race and classism, race and homophobia, race and ageism, and race and ableism. Narrative analyses of these reports were conducted.

### Statistical Analysis

Descriptive statistics were used to analyze survey data, including frequencies, percentiles, means, SDs, medians, IQRs, and overall ranges. SPSS version 29 (IBM Corp) was used. Classical content analysis was used for focus group and narrative analyses of final reports. Here, independent open and axial coding of field notes was undertaken using constant comparative analysis to identify emergent themes.^17^ Consensus meetings were used to review codes, develop descriptions of emergent themes, and select exemplars. An interpretivist philosophical paradigm was used, where investigators described human experiences through a lens of culturally derived and historically situated explanations of the world.^18^

## Results

Forty-two proposals were received from across the institution, and 17 (40.4%) were selected for funding. Characteristics of the 19 recipients demonstrated active participation from those historically minoritized by race (12 [63.2%]; ie, 5 [26.3%] Asian; 2 [10.5%] Black or African American; 1 [5.3%] Hispanic or Latinx; 4 [21.1%] Middle Eastern, North African, or biracial; and 7 [36.8%] White), sexual orientation (6 [31.6%]; 1 [5.3%] bisexual; 3 [15.8%] gay; and 2 [10.5%] lesbian), and disability status (3 [15.8%]) (Table 1). Fifteen participants (78.9%) were women, and 1 (5.3%) was genderqueer or gender nonconforming. All projects (100%) responded to the survey at time 1. Two projects were overrepresented due to multiple responses from the same proposal (thus n = 19 rather than 17). Table 2 describes the diverse nature of the projects, funding allotted, and number of participants.^19^ The majority of funded projects (14 of 17 [82.4%]) were based in the medical school. One recipient (5.6%) declined to receive funding after realizing they lacked the capacity to successfully execute their proposed project, 3 more (16.7%) returned the funds after attempting to implement, and 2 (11.1%) requested extensions due to unforeseen events. Eleven projects were implemented using $157 601.50 or 78.8% of available funds (Table 3),^19,20,21,22,23^ and $42 389.50 or 21.2% of funds were returned. A total of 1741 individuals were engaged during the funding period (Table 2).

**Table 1.** Characteristics of Designated Team Members

Participant Characteristics	Participants, No. (%)^a^
Role on the study	
Principal investigator	13 (72.2)
Study team member	5 (27.8)
Gender identity	
Woman	15 (78.9)
Man	3 (15.8)
Genderqueer or gender nonconforming	1 (5.3)
Sexual orientation	
Bisexual	1 (5.3)
Gay	3 (15.8)
Heterosexual or straight	13 (68.4)
Lesbian	2 (10.5)
Age, y	
<30	3 (15.8)
30-49	10 (52.6)
≥50	6 (31.6)
Racial and ethnic identity	
American Indian or Alaska Native	0
Asian	5 (26.3)
Black or African American	2 (10.5)
Hispanic, Latino, or Spanish origin	1 (5.3)
Native Hawaiian or Pacific Islander	0
Middle Eastern, North African, or biracial	4 (21.1)
White	7 (36.8)
Primary language	
English	17 (89.5)
Spanish	2 (10.5)
School or college affiliation	
Dentistry	1 (5.9)
Medicine	14 (82.4)
Nursing	2 (11.8)
Pharmacy	0
Public health	0
Disability^b^	
Yes	3 (15.8)
No	16 (84.2)

^a^ The total varies from 17 to 19 because 2 unique participants on 2 teams responded to the survey.

^b^ Including but not limited to physical disability, intellectual disability, learning disability, chronic illness disability.

**Table 2.** Mini-Proposals Awarded to Address Antiracism at Oregon Health & Science University

Proposal No.	Title	Description	School, department. or unit	Budget used during grant period
1	Al-Shafaa Mobile Dental Clinic REI Funding Proposal	Weekly mobile dental clinic in Portland, OR and Vancouver, WA Volunteer dentists, physicians, and students with ties in the Muslim community provide care to underrepresented populations	School of Dentistry; student led	Unable to use funds
2	Bias Equity and Inclusion in Artificial Intelligence for Medical Systems Internship	Internship program for undergraduate URM students Students are mentored while participating in research projects developed within the Artificial Intelligence for Medical Systems (AIMS) Lab	School of Medicine; Biomedical Engineering	$20 000.00
3	Black Like Me 360: A Virtual Experience	Collaboration between the Black Employee Resource Group and the National Association for the Advancement of Colored People (Portland chapter) Black actors interact with White persons wearing a 360° GoPro Max camera to reenact true negative race-related interactions that represent day-to-day lived experiences	School of Medicine; Biomedical Engineering	Unable to use funds
4	Building a Trauma-Informed Education Resource Center: A DEI OHSU collaborative	Develop a Trauma-Informed Educator Resource Center with a focus on DEI Educators use the learning environment to explore how racism and oppression operates at OHSU Faculty and administrators learn how to disrupt harmful narratives to prevent trauma across campus	School of Nursing, Monmouth Campus	$6494.50
5	Developing a program to encourage and support innovation by BIPOC and women	Develop a Trauma-Informed Educator Resource Center with a focus on individuals from minoritized racial and ethnic groups and women Educators use the learning environment to explore how racism and oppression operates at OHSU and how to disrupt harmful narratives to prevent trauma cross campus	Institution-wide^a^	$10 000.00
6	Diversifying IRB Membership and Education	Develop a program to recruit faculty and community at-large members who belong to minoritized racial and ethnic groups to OHSU’s IRB Foster dialogue about research experiences and inform research that is inclusive and racially appropriate New members could assist with recruitment of diverse study participants, avoid continued trauma of underrepresented groups, rebuild participant trust in research processes, and promote greater access to research	Institution-wide^b^	Unable to use funds
7	Diversity Awareness Through the Arts and Culture	Develop inclusion, equity, and diversity awareness among educators, staff and students through performing arts and culture Include local nonprofit organizations, nationwide artists, and guest speakers to create a supportive and respectful learning Invited artists or keynote speakers will present different perspectives on art and cultural topics	Education Improvement and Innovation	$10 000.00
8	Diversity, Equity and Inclusion (DEI) Workshops for Healthcare Professionals	Develop DEI and antiracism workshops for health care professionals with support and endorsement of the Graduate Medical Education Multi-Specialty Committee Provide focused topics and resources surrounding DEI applicable to any residency program Educate trainees and create reflection and discussion processes about current and future DEI efforts	School of Medicine, Urology; Ophthalmology	Unable to use funds
9	Experiential Learning in Qualitative Research and Mentorship in Biomedical Research and Medicine for Underrepresented Minority (URM) Students	Develop an internship program for students in public high schools and undergraduate colleges with high representation of PEER in science, including Black, Hispanic, and female students Students participate in a program entitled, “Understanding the impact of COVID-19 on faculty research productivity in the Department of Surgery”	School of Medicine, Vascular Surgery	$10 000.00
10	Increasing Diversity Images in Materials for Learners, Employees, and Patients at OHSU and Beyond	Increase images representing diversity and intersectionality used in OHSU curricula Create and curate a collection of new images in a library database Provide workshops for faculty and staff to select images for OHSU mission related purposes	School of Nursing; Library	Unable to use funds
11	Leveraging an Existing Program to Improve Racial Equity & Inclusion Competency at OHSU & Beyond	Expand a successful, well-established program (Novel Interventions in Children’s Healthcare) to highlight and center patient and family voices for staff training Expanded topics include identifying barriers to care and systemic inequities that address the intersectionality of race, sexism, classism, homophobia, and ageism	School of Medicine, Psychology	$1950.00
12	Needs Assessment for Black and Latinx Parents in the NICU	Develop an education program for faculty, staff, and students on disparities that Black and Latinx families’ experience in the NICU Establish and promote a culture of equity and antiracism with a focus on infants with complex medical needs Develop a culture of trust in a place where families begin long-lasting relationships with the medical system	School of Medicine, Pediatric Neonatal Intensive Care	$9057.00
13	Rad Young Portlanders	Create a high school and middle school outreach program to increase socially and economically underrepresented youths’ interest in and awareness of health care and STEM Reduce health disparities and create a more diverse medical workforce	School of Medicine; Diagnostic Radiology	$10 000.00
14	Telehealth and the Digital Divide: Identifying Potential Care Gaps in Virtual Visit Use	Project to address the discrepancy between race, age, and insurance status on telehealth utilization Use a human-centered design approach to create tools to close the digital divide and narrow gaps in health disparities	School of Medicine, IT Health Care Applications	$2100.00
15	The “Power With” Anthology: Storytelling for Health Justice	Engage the OHSU community by developing a collection of multimedia stories that highlight the voices of those with lived experiences in the health care system Use the collection in facilitated community discussions Stories will be collected from those traditionally oppressed by “power-over” hierarchies	School of Medicine; Family Medicine	$10 000.00
16	Toward Equity and Inclusion in Peer Review Exploring Structural Bias Embedded in Health Research	Project to create a transparent, equitable peer review process that reduces implicit and explicit bias Support outreach efforts, including focus groups and consensus forums The final product of this project will be a report and recommendations	School of Medicine; Department of Medical Informatics	Unable to use funds
17	Speaker Series for Health Equity & Anti-Racism	Project designed to implement a collaborative institution-wide speaker series on REI.^19^	Institution-wide^c^	$68 000.00

Abbreviations: BIPOC, Black, Indigenous, and Person of Color; DEI, Diversity, Equity, and Inclusion; IRB, institutional review board; NICU, neonatal intensive care unit; OHSU, Oregon Health & Science University; PEER, persons excluded due to ethnicity or race; REI, Race, Equity, and Inclusion; STEM, science, technology, engineering, and mathematics; URM, underrepresented in medicine.

^a^ Initiated by Tech Transfer Office.

^b^ Initiated by OHSU’s IRB.

^c^ Initiated by Otolaryngology, Research & Innovation Office, Health Plan Services, Dermatology, Psychology.

**Table 3.** Funding Allocations, Status, Outcomes, Barriers, and Community Engagement

Proposal No.	Proposal title	Funding allocation	Status at end	Outcomes	Barriers	Individuals engaged during funding period^a^
1	Al-Shafaa Mobile Dental Clinic REI Funding Proposal	Funds not used	NA	NA	Operational logistics and bandwidth (FTE)	2
2	Bias Equity and Inclusion in Artificial Intelligence for Medical Systems Internship	Learner stipend	Internship program started with 3 interns of diverse backgrounds, 2 presentations on work	Dissemination of scholarship, diversity recruitment	Access to databases	5
3	Black Like Me 360: A Virtual Experience	Funds not used	Ongoing^20^	Website template created	Participation from partners	1
4	Building a Trauma-Informed Education Resource Center: A DEI OHSU collaborative	Learner stipend	Website developed and materials available^21^	Professional development (enduring material)	Bandwidth (FTE)	2
5	Developing a program to encourage and support innovation by BIPOC and women	Subscription to online diversity training, honoraria	Developed reporting tools for disaggregation, professional development	Professional development (limited)	Participation from partners	49
6	Diversifying IRB membership and education	Funds not used	Recruited 3 new diverse IRB members, developed a new recruitment process, culturally appropriate tools	Diversity recruitment, policy or procedural change	Participation from partners, bandwidth (FTE), virtual environment	79
7	Diversity Awareness Through the Arts and Culture	Honoraria	Increased appreciation of diversity, fostering understanding, and exposure	Professional development (limited)	Operational logistics and participation from partners	228
8	Diversity, Equity, and Inclusion (DEI) Workshops for Healthcare Professionals	Funds not used	NA	Professional development (enduring material)	Participation from partners	3
9	Experiential Learning in Qualitative Research and Mentorship in Biomedical Research and Medicine for Underrepresented Minority (URM) Students	Learner stipend	Increased understanding of the impact of the COVID-19 pandemic on its clinical research workforce	Dissemination of scholarship, diversity recruitment	Operational logistics	9
10	Increasing Diversity Images in Materials for Learners, Employees, and Patients at OHSU and Beyond	Funds not used	Website materials available^22^	Professional development (enduring material)	Participation from partners, bandwidth (FTE)	3
11	Leveraging an Existing Program to Improve Racial Equity & Inclusion Competency at OHSU and Beyond	Incentives and honoraria	Reflected on and discussed barriers to care and systemic inequities	Dissemination of scholarship	Participation from partners	16
12	Needs Assessment for Black and Latinx Parents in the NICU	Incentives and honoraria	Convened 2 community advisory boards, 1 for Black and 1 for Latinx families and collaboratively developed the culturally specific focus group/interview guide	Policy or procedural change	Rigid timelines, limited funding, operational logistics	38
13	Rad Young Portlanders	Portable ultrasounds and other teaching materials	Partnered with Benson Polytechnical High School’s health occupations academy. Through lectures and workshops, these high school students have a better sense of the different career opportunities with the field of radiology, the various subspecialties within radiology, and how they fit within health care; 7 total presentations to date	Diversity recruitment	Bandwidth (FTE)	159
14	Telehealth and the Digital Divide: Identifying Potential Care Gaps in Virtual Visit Use	Development of prototype	Created a potential model at OHSU for how patients and community-based organizations can be directly involved in design and research processes and have legitimate power and influence in addressing gaps in care.	Dissemination of scholarship	NA	3
15	The “Power With” Anthology: Storytelling for Health Justice	Incentives and videography	Website materials available^23^	Professional development (enduring material)	Operational logistics and bandwidth (FTE)	665 views
16	Toward Equity and Inclusion in Peer Review Exploring Structural Bias Embedded in Health Research	Funds not used	NA	NA	Bandwidth (FTE) and operational logistics	2
17	Speaker Series for Health Equity & Anti-Racism	Honorariums	Website materials available^19^	Professional development (enduring material)	Bandwidth (FTE) and operational logistics	477

Abbreviations: BIPOC, Black, Indigenous, and Person of Color; DEI, Diversity, Equity, and Inclusion; FTE, full-time equivalent; IRB, institutional review board; NA, not applicable; NICU, neonatal intensive care unit; OHSU, Oregon Health & Sciences University; REI, Race, Equity, and Inclusion; URM, underrepresented minority.

^a^ A total of 1741 individuals were engaged, with a range of 1 to 665 for the projects.

Table 3 illustrates funding allocations, status, outcomes, barriers experienced, and level of community engagement. Funds were primarily used for incentives, learner stipends, honoraria, or project materials. Barriers to implementation commonly included limited bandwidth, lack of partner participation, and organizational and logistical issues. While some projects produced enduring and likely self-sustaining materials (defined by participants), especially for educator development or changes in policy, other projects were one-and-done.

Ten projects participated in the community engagement survey (58.8%), reflecting the intention to engage community partners at all 4 levels of engagement: inform (6 activities), consult (10 activities), collaborate (21 activities), and enable (5 activities). The intended and actual community engagement levels included 633 individuals, 22 departments, and 4 schools. Projects within this study reported engaging their intended audiences in their final reports (eFigures 1 and 2 in Supplement 1).

Two focus groups were attended by a representative from 7 projects (41.1%). Analyses of the focus group and final report data revealed that 8 of 11 projects (72.7%) where funding was not returned achieved implementation (eg, self-reported use of funding and engagement of intended participants) and 3 did not. Emergent themes expressed by participants while planning and implementing their projects included (1) timing of the project rollout; (2) preparedness challenges; (3) distrust from the community due to previous diversity and inclusion experiences; (4) support from peers; (5) improved well-being for participants; and (6) difficulties breaking down institutional systems (Box). Participants expressed both conflict and dissonance, as they had hoped for more positive systematic institutional change even while recognizing existing institutional barriers. Few successful participants indicated that an impact had been seen in their intended audiences and participating staff. The early systematic barriers delayed many projects and in turn, prolonged the ability to observe long-term influence. In contrast to the short-term barriers, some awardees revealed that they found community support while implementing their projects, giving them hope for continued diversity and inclusion opportunities at OHSU and eventual long-term influences.

Box. **Themes and Qualitative Evidence**

**Timing of the project rollout**

“It’s been challenging to, to say the least, and it, it’s, it’s also been quite a … because we’ve only had a very short period of time to work on this too. That has also been a challenge, which I’m sure many of you can relate to.” (P4/G1)
“The planning of it was very rushed, because the um, I know that there was a like, a 1 month extension for, um, the application process for the grant, but I didn’t feel like I had the chance to strategize really? And collect some of the things that I needed to collect before I applied.” (P1/G1)
“So, my, you know, my project, we haven’t launched the platform yet. Um, we, we didn’t realize that, um, the admin assistant had to do training and then she to wait for to sign up for the training, like there was a wait period or whatever.” (P1/G1)
“There are external forces of our project that are very eager for us to have something done right now and trying to bounce their needs with ah, the needs of the groups that we’re actually, you know, trying to target and help, um, it has been a challenge.” (P4/G1)

**Preparedness challenges**

“Really had a hard time with recruitment.…To a broader more diverse population is really a first step. Um, so that that’s kind of what, what I’m learning the hard way.” (P3/G1)
“I wish going into this program that we had received additional support, um…work on a proposal that is trauma informed. That addresses cultural competency and cross-cultural communication…. I think we thought we were further along when we started than we really were.” (P4/G1)
“I don’t know that we’re ever prepared for this, or the work in terms of diversity, equity inclusion, um, because I don’t think that we’ve been doing it. We don’t have we don’t have sort of a blueprint.” (P1/G1)
“We want to make sure that we don’t continue the perception in fact, in in the past OHSU of being unable to listen and do right by the community. And so, to shift that it takes a lot of intentionality and many groups of people that come together and you know, if we had a limited time, or we didn’t do anything else, then it could move more quickly, but to make sure we have the right people in those conversations that there’s enough space to breathe and really, talk through some of these immediate issues it.” (P1/G2)
“Like, we don’t want to just, like, check a box of, like, here’s a certain type of person that we’re hoping to represent in this story collection. So sort of. So, anyway, we kind of like, switch the process of how we might find potential storytellers and I feel like there’s this really fine line, um, around making sure that we are lifting at voices that aren’t traditionally heard, um, in this space.” (P3/G2)

**Distrust from the community due to previous diversity and inclusion experiences**

“There’s a lot of suspicion around these kind of projects, too that I have found with folks that we have engaged with who are concerned….Is this performative is this, like, are you really trying to do something here and…Ah, I, I was not expecting to be having those conversations.” (P3/G1)
“I needed to recruit black people basically of OHSU, and this is where I got really stuck because what I’m seeing is that black people at don’t trust things going on at OHSU.” (P4/G2)

**Support from peers**

“One thing that has been a surprise, but in a great way, is how many people want to be a part of these sort of initiatives and are excited and passionate about it.” (P4/G1)
“I’ve been amazed at how many people are engaged in wanting to do this work and we regularly have we have weekly team meetings as part of this … you know, clinicians staff, um, clinic based staff, it’s been really awesome. I think I’ve been surprised at how engaged, particularly the providers on the team, because their schedules are just ridiculously busy.” (P1/G2)
“Bring voice and agency to patients into a design process really is resonating with the culture we have at OHSU, in and I think it provides an actionable way for people to, um…for us, the design systems that work better. Um, so that’s been for me a surprise. A pleasant surprised at how engaged everyone is.” (P1/G2)
“I just wasn’t expecting people to be as excited about it as I was. And so it was really a nice surprise. Like, I’m not the only 1 who’s into this. I’m not the only 1 who thinks that this could actually make a difference that this could actually get some kind of message across.” (P4/G2)
“Um, and I was surprised by the level of support that we got from family medicine, um, and broadly at OHSU.” (P3/G2)
“I don’t know if others share this, but for me, having the institution that has, you know, some credibility like, OHSU say, hey, like, this work matters and we’re going to fund projects.” (P1/G2)
“I have to agree with that right now that there’s something about getting this grant. And getting confirmation that the university cares about this. Uh, subject as much as I do is really affirming.” (P4/G1)

**Improved well-being for participant**

“Is it’s just a huge boost of, like, wow, okay. Is really at least for this period of time, they’re like acknowledging, they’re validating that this is important work. Um, so, for me, personally, that was really a huge boost and and I see potentially a path forward to continue doing this kind of work in an institution, like, which is more focused on research in academia, um, so that’s all super exciting for me. I’m hoping this will be a kind of a springboard to continue doing more of that work here.” (P2/G2)

**Difficulties breaking down institutional systems**

“When I’m talking about, um…um. you know, implementing trauma, informed, educational practices people, are on board, you know, but, you know, they, there is so much pushback to really changing our structures. It’s so hard for people to really see past um, the way we’ve been doing things and to really open themselves up to the recognition of, of what it’s going to take.” (P1/G1)
“That younger population wanting to activate and make these changes but it’s also, on the other hand, I guess sad that, um, some of the more experienced researchers, um, may not. I don’t know if it’s a lack of hope or, you know, or think they think things can’t change. Um, I mean, I’m hoping to get more so that we can talk about it and we’ll actually find out what some of these barriers are.” (P3/G1)
“It speaks to the immaturity of our system, in bringing up and how our systems do or don’t show the stories or create moments where we can understand one lens of data into the stories, of at least from what our patients are experiencing and who they are, even more than that, who are they?” (P1/G2)

Abbreviations: G, group; OHSU, Oregon Health & Science University; P, participant.

Additionally, while the funding opportunity did not set out to influence well-being and a sense of purpose at the start of the project, these themes emerged in focus group data as unintended outcomes. Participants mentioned feeling energized by receiving funding for their projects, as a reaffirmation that their work is important to their peers and the university, which counters the typically assumed “cultural tax” that often occurs with minoritized individuals. Some participants mentioned that funding opened new opportunities and relationships across the institution.

## Discussion

The REI funding opportunity program was implemented with a clear and intentional plan to select, retain, and ensure the success of awardees. Focus group data suggested that implementation fidelity of the funding opportunity program was strongest at the selection phase of awardees. As awardees implemented their projects independently in their local settings, they reported a range in levels of engagement, quality of programming, and exposure of the project to institutional members. However, not all funded teams were able to implement their intended program during the funding period. Four returned all their awarded funds, leaving over $40 000 unspent. Seven projects requested funding extensions to complete their work. This suggests that even with careful thought and preliminary planning, some challenges were insurmountable (eg, staff support, distrust of the community in institutional efforts).

The intended goals of the program were to (1) encourage faculty, staff, and students to be actively involved in reducing racism and the intersectionality of race and sexism, race and classism, race and homophobia, race and ageism, and race and ableism; (2) promote antiracist practices in recruitment and retention efforts in educational programs at OHSU; and (3) support the development of educational initiatives and policies that value humanity, promote antiracism, and allow for all voices to be heard, valued, and recognized. These goals were aspirational in nature, as the antiracist policy and procedural work did not occur at the pace and timeline envisioned for all proposals. While 35% of grantees were not able to use the funding during the time allotted, 65% of grantee-funded projects reached more than 1700 participants, and enduring material or diversity initiatives were created, which will continue to reach more participants and have ongoing institutional influence. The inability to measure the influence of the grants before the end of the 1-year grant period was not surprising, as many awardees’ initiatives during the focus group were still in their infancy.

Several barriers identified were considerable and were not malleable.^24^ Many fundees had difficulty finding time to undertake their projects in their already busy schedules. Because full-time equivalent (FTE) support was not allowed to be covered by available funds, projects were added onto established workloads, which was not optimal. The bandwidth issue also likely contributed to challenges with operational and logistical issues as well as with engaging partner participation. While we hoped that these projects could lead to disruptive innovation, it appears likely that the amount dedicated to this effort was insufficient to even begin to create sustainable change. Institutions seeking to initiate efforts like this should allow FTE support to be covered by the effort and should ensure that funds available are dedicated to enduring efforts rather than single one-and-done programs. This is important, given current literature suggests that the higher level of community engagement, the higher potential for cultural change across an institution.^16^ Community engagement is complex and success has been noted by following community-based participatory research principles, including (1) recognizing the community as a unit of identity, (2) building on the strengths and resources of the community, (3) promoting colearning among research partners, (4) achieving a balance between research and action that mutually benefits both science and the community, (5) emphasizing the relevance of community-defined problems, (6) using a cyclical and iterative process to develop and maintain community/research partnerships, (7) disseminating knowledge gained from the community-based participatory research project to and by all involved partners, and (8) requiring long-term commitment on the part of all partners.^25^

These antiracism funding opportunities now are ending, and there is still much work to be done. Future initiatives should allocate FTE support to those leading antiracist projects; invest in more mentorship throughout the project; support marketing and dissemination activities; and ensure that each project has an active institutional sponsor to help each project navigate barriers throughout the program and not just at the beginning. Sponsorship, while essential, was a voluntary commitment. Future funding opportunities should consider the potential influence of offering incentives for participation and supporting enduring efforts rather than one-and-done approaches. Lastly, longitudinal efforts with more robust study designs are needed to fully assess the results of initiatives such as this.

### Strengths and Limitations

The strengths include the diversity in funded projects as well as the diversity in members of the project teams. We think the unique approach we undertook to inspire cultural change through racial equity mini-funding opportunities has some limitations. First, this report is a summary of work done at a single institution. Second, while the authors examined the intended community engagement of participants, the actual community engagement through the funding opportunities was not fully captured. Future initiatives like this should distinguish between intended vs actual outcomes. Third, this is a meta-program evaluation of the administrative procedures and policies of funding opportunities rather than the implementation of each funding opportunity. As a result, we were unable to determine how much each funded project led to antiracist cultural change, but we were able to demonstrate the intentional institutional influence, program self-evaluation, and implementation fidelity. Future studies should examine the antiracist administrative procedures and policies at the planning phase of activities but also the implementation and evaluation procedures and policies.

We believe initiatives like this one can provide opportunities to reinforce the recommendations from Argueza et al^1^ for addressing racism in intentional ways. We hope that our work will serve as an example of disruptive innovation that can steadily change institutional culture. As noted in the literature, the neoliberal and capitalist approaches to efficiency that are foundational to the structure of academic medical centers and higher education can often be in contradiction with trauma-informed, equity, and social justice work on campuses.^26,27^ The antiracist environments that institutions desire must be engaged in more than a transactional passing of the baton to its members to do anti-racist work, but a different way of organizing and carrying out day-to-day work with a sensitivity to power and privilege and capacity to facilitate equitable outcomes.

This study has limitations. We are not certain we reached thematic saturation with the 2 focus groups conducted. This may result in not fully identifying all themes that affected the execution of this work. The challenge of addressing and supporting systematic antiracist change in academic medical institutions is met with pervasive barriers that are not unique to this institution but are shared across the globe. This study was carried out in a single academic environment, and while that may be a limitation, structural racism exists in many academic health centers. More research in this area is needed before generalizability can be addressed in unique environments. In fact, our study may raise more questions than it answered. While individuals are committed to caring and thinking deeply about action, how do organizations improve operations and daily work in systematic ways that allow individual and group efforts in diversity, equity, inclusion, and antiracism to be successful? This will require robust planning and implementation that include the individual and communal promotion of relationships, earning trust, supporting healing, and the desire to grow, unlearn, relearn, and change. More explicitly, antiracist funding opportunities should document how they prioritize and establish community partnerships as part of the planning and selection process. Timelines for implementation must be flexible enough to allow participants to acknowledge barriers, identify sponsors, support solutions, and revise their approach as they move toward their aspirational goals. To this end, each reader is a potential contributor to both barriers and solutions. How do you and how will you support this work?

## Conclusions

In this study, 17 antiracism proposals were selected for funding, led by a diverse group of participants. A total of 11 projects were implemented, engaging a total of 1741 individuals. Participants identified barriers to successful implementation and also reported improved well-being and support from peers.

## Notes

Supplement 1. **eFigure 1.** Frequency of Intended Community Engagement by Level **eFigure 2.** Frequency of Intended Engagement of Community Partners **eAppendix.** Grant Proposal Review Rubric (Pre-Proposal Review)

Supplement 2.
Data Sharing Statement
