# Supplementary material for: Funding Opportunities Designed to Promote Antiracist Change Across a Health Sciences University
Source: JAMA Netw Open. 2023 Oct 10;6(10):e2337096. doi: 10.1001/jamanetworkopen.2023.37096 (PMC10565608; doi:10.1001/jamanetworkopen.2023.37096)
Supplement: Supplement 1. — eFigure 1. Frequency of Intended Community Engagement by Level eFigure 2. Frequency of Intended Engagement of Community Partners eAppendix. Grant Proposal Review Rubric (Pre-Proposal Review) [file jamanetwopen-e2337096-s001.pdf]

Supplemental Online Content

Tucker CR, Lahti E, Carney PA. Funding opportunities designed to promote antiracist change across a health sciences university. *JAMA Netw Open.* 2023;6(10):e2337096. doi:10.1001/jamanetworkopen.2023.37096

eFigure 1. Frequency of Intended Community Engagement by Level

eFigure 2. Frequency of Intended Engagement of Community Partners

eAppendix. Grant Proposal Review Rubric (Pre-Proposal Review)

This supplemental material has been provided by the authors to give readers additional information about their work.

© 2023 Tucker CR et al. *JAMA Network Open.*

eFigure 1. Frequency of Intended Community Engagement by Level (N=10)

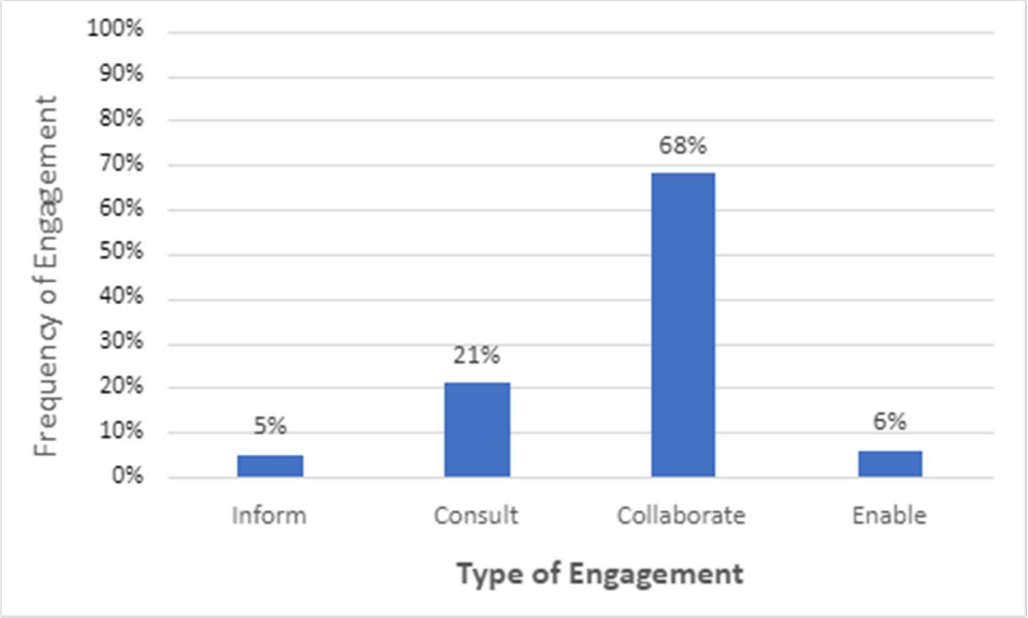

Self-reported level of stakeholder engagement across all funding opportunities. Categories not mutually exclusive.

© 2023 Tucker CR et al. *JAMA Network Open.*

eFigure 2. Frequency of Intended Engagement of Community Partners (N=10)

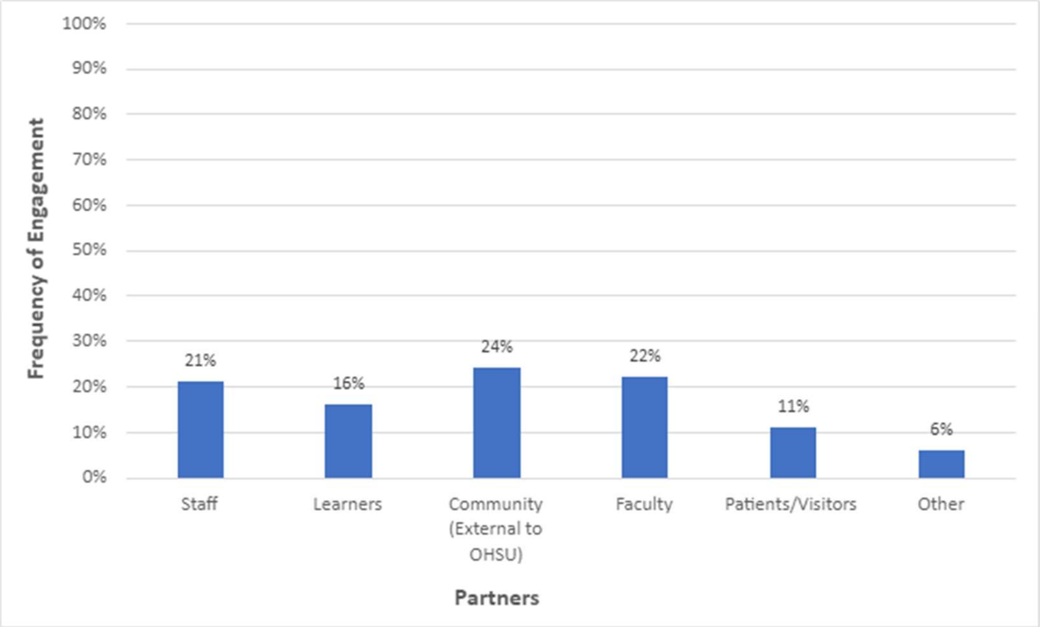

Level of engagement across diverse types of community partners. Categories are not mutually exclusive.

© 2023 Tucker CR et al. *JAMA Network Open.*

eAppendix. Grant Proposal Review Rubric (Pre-Proposal Review)
PROJECT TITLE: _

Criterion	Strong	Satisfactory	Developing
**Alignment with grant program goals**: The project identifies an area for improvement in the program's learning outcomes	This proposal identifies an area for improvement that, if addressed, will significantly improve, stimulate and sustain anti-racist behaviors and outcomes while acknowledging intersectionality.	This proposal identifies an area for improvement that if addressed may improve anti-racist behavior.	It is unlikely that this proposal if addressed will significantly improve stimulate and sustain anti-racist behavior.
**Impact and Importance**	The planned action(s) directly respond to and will improve institutional systems (i.e., procedures, policies, social determinants of health, etc.)	The planned action(s) directly respond to and will improve OHSU member knowledge and performance	The planned action(s) engage participation and satisfaction of OHSU members
**Collaboration**: Multiple stakeholders in the program have an active role in carrying out the proposed project.	Multiple stakeholders (including community members) in the program have an active role in carrying out the proposed project, thereby increasing its impact.	Multiple stakeholders (including community members) in the program have agreed to participate but have a limited role in carrying out the proposed project, thereby limiting its impact.	A single stakeholder will carry out the proposed project with limited participation from or impact on other stakeholders
**Timeline**: The project can be completed within the proposed timeline	The project can be completed within the proposed timeline	It will be challenging to complete the project within the proposed timeline without additional help, expertise or funds	It is unlikely that the project can be completed within the proposed timeline.

SUMMARY RECOMMENDATION

**Recommendation for funding:** Based on the above elements together, the project is likely to achieve its stated goals for promoting best practices.	**Fund this proposal:** Most elements received a "Strong" rating; any elements with a "Satisfactory" rating can be easily improved. (Add feedback below.)	**Consider this proposal:** Some elements were rated "Strong," but others were rated "Satisfactory," indicating some areas that need further development. With feedback, the proposers could improve the project plan. (Add feedback below.)	**Do not fund this proposal in its current form:** The proposal does not align with the goals, or several of the elements above were rated as "Developing." With feedback, the proposers could revise their project plan for consideration in future grant cycles. (Add feedback below.)

Feedback to proposers:

© 2023 Tucker CR et al. *JAMA Network Open.*
