# Supplementary material for: Funding Opportunities Designed to Promote Antiracist Change Across a Health Sciences University
Source: JAMA Netw Open. 2023 Oct 10;6(10):e2337096. doi: 10.1001/jamanetworkopen.2023.37096 (PMC10565608; doi:10.1001/jamanetworkopen.2023.37096)
Supplement: Supplement 2. — Data Sharing Statement [file jamanetwopen-e2337096-s002.pdf]

Data Sharing Statement

Tucker. Funding Opportunities Designed to Promote Antiracist Change Across a Health Sciences University. *JAMA Netw Open*. Published October 10, 2023. doi:10.1001/jamanetworkopen.2023.37096

Data
Data available: No

Additional Information
Explanation for why data not available: Data collected for this study were approved by IRB as confidential: identification of participants poses risk to the participants (i.e., employees, students, and community members). De-identified data files, including statistical syntax, data dictionary and qualitative data and coding are available upon request.
